# Relationship between Simulated Fire Suppression Activities and Acute Cardiac and Respiratory Events in Firefighters

**DOI:** 10.3390/jfmk9020096

**Published:** 2024-05-31

**Authors:** Roger O. Kollock, William D. Hale, Maddie Fulk, Maddie Seidner, Zora Szabo, Gabriel J. Sanders, Will Peveler

**Affiliations:** 1 Department of Kinesiology and Rehabilitative Sciences, The University of Tulsa, Tulsa, OK 74104, USA; wdh1231@utulsa.edu (W.D.H.); mlf7706@utulsa.edu (M.F.); mjs9787@utulsa.edu (M.S.); zls9345@utulsa.edu (Z.S.); 2 Exercise Science Department, University of Cincinnati, Cincinnati, OH 45221, USA; sandergj@ucmail.uc.edu; 3 School of Health Sciences, Liberty University, Lynchburg, VA 24515, USA; wpeveler@liberty.edu

**Keywords:** injury, physical abilities, casualty, overexertion, aerobic capacity, VO_2_max

## Abstract

Cardiac deaths account for the largest share of on-duty firefighter deaths. To help ensure duty fitness and minimize injury risk, many fire departments require the passing of an annual physical ability test, consisting of a battery of simulated fire suppression activities (sFSAs). The purpose of the study was to determine the relationship of sFSA performance to acute cardiac and respiratory events (ACREs) and the effect that estimated VO_2_max has on sFSA performance. The study was retrospective. As part of an annual physical ability test, five timed sFSAs were performed, summed for a composite time, and categorized into three performance levels (fast, moderate, and slow). Estimated VO_2_max was determined using the Forestry Step Test. A significant (*p* = 0.023) linear trend was observed with higher sFSA performance times being associated with a higher proportion of firefighters going on to suffer an ACRE. The estimated VO_2_max was significantly (*p* < 0.001) higher in the fast group compared to the slow group. There was not a significant (*p* = 0.70) difference in estimated VO_2_max between the moderate and slow groups. Estimated VO_2_max performance and sFSA performance were significantly correlated, with r_s_(488) = −0.272 and *p* < 0.001. Poorer sFSA performance was found to be associated with a higher proportion of ACREs. The results suggest that sFSA performance may be a valid indicator of ACRE injury risk and aerobic capacity.

## 1. Introduction

Fatal and nonfatal casualties represent a significant “burden” to those involved in fire and rescue. The mechanical and physiological stress placed on the body, due to the extreme occupational demands of firefighting, is likely one key contributor to stress or overexertion being the leading cause of fireground casualties [1,2]. In 2021, there were 141 reported firefighter fatalities in the United States, with 39 resulting from stress or overexertion [3]. While there was a reduction in fatalities in 2022 (*N* = 96), 51% were a result of stress or overexertion, with 35% of those identifying the cause of death as sudden cardiac failure [4]. Sudden cardiac deaths or cardiac conditions regularly account for the largest share of on-duty firefighter deaths [4]. As of 2022 in the United States, stress or overexertion represents 32% of all injuries sustained on the fireground [5].

There is at least some evidence suggesting the importance of adequate cardiorespiratory fitness [6] and other components of fitness (e.g., muscular strength, flexibility, muscular endurance, and healthy body composition) [7] in minimizing a firefighter’s overall casualty risk [6,7]. In general, it has been observed that firefighters with a lower comprehensive fitness status were 1.81 times more likely to sustain an injury [7]. However, these earlier findings are not specific to injuries such as acute cardiac and respiratory events (ACREs) caused by stress or overexertion. To help ensure their incumbents are fit for duty and at minimal risk for injury, many municipalities and state agencies require the passing of an annual physical ability test, which consists of a battery of simulated fire suppression activities (sFSAs). To date, there is no standard across fire departments in the administration of physical ability tests (e.g., events included and scoring or timing of the events). The sFSAs included in the physical ability test often are variations of events used on the Candidate Physical Ability Test. The Candidate Physical Ability Test was designed to allow fire departments to obtain pools of trainable candidates who are physically able to perform essential job tasks at fire scenes [8]. The events included in a Candidate Physical Ability Test include (1) stair climb, (2) hose drag, (3) equipment carry, (4) ladder raise and extension, (5) forcible entry, (6) search, (7) rescue, and (8) ceiling breach and pull [8,9]. The Candidate Physical Ability Test is a pass-or-fail test based on a maximum total time of 10 min and 20 s [9]. Although some fire departments use Candidate Physical Ability Test style events or other types of sFSAs as a means of determining physical readiness, there is limited evidence that completing a battery of sFSAs within a given clock time lends to a reduced risk of injury or ACRE.

For most FSAs, firefighters must work in a high range of cardiorespiratory capacity, for an extended amount of time with limited recovery [10]. There is at least some evidence that suggests a relationship between fire-suppression activity (FSA) performance and cardiorespiratory fitness [11]. The continuous exertion at this level requires efficient economical movement patterns. The cardiorespiratory strain at such intensities creates a significant risk factor for an ACRE in those firefighters with lower levels of fitness [12]. Kales and colleagues [13] determined that firefighters are at least 10 times more likely to suffer an ACRE during an FSA than a nonemergency task. To combat this risk, the National Fire Protection Association recommends fire service incumbents maintain a cardiorespiratory capacity of ≥42 mL/kg/min for optimizing cardio-protection [11]. The recommendation of ≥42 mL/kg/min by the National Fire Protection Association, in many cases depending on the individual’s age, may not be consistent with that of the American College of Sports Medicine. For example, younger individuals between the ages of 20 and 29 to be classified as good (60th percentile) would need a VO_2_max of 50.2 mL/kg/min, while those between the ages of 30 and 39 require a VO_2_max of 45.2 mL/kg/min according to American College of Sports Medicine guidelines [14]. Note that this range set by the American College of Sports Medicine indicates an acceptable (protection from ACREs) cardiorespiratory capacity for adults who are not charged with fire suppression activities. A cardiorespiratory capacity below these acceptable levels places individuals at significant risk for an ACRE [14].

Due to the average duration of FSAs, 20–45 min repeated 2–4 times per event, and short rest interval (5–10 min), there is a reliance on both aerobic and anaerobic (glycolytic) sources of energy [15]. In a 2022 study, Beitia and colleagues [16] reported that 35–60% of metabolic demands during structured firefighting activities come from anaerobic energy production due to the extended time working at 97% of their VO_2_max. A study by von Heimberg et al. [17] noted that, due to the limited volume of air contained within SCBA (self-contained breathing apparatus) devices, firefighters who can complete tasks faster would conceivably consume less air, indicating a need for a developed anaerobic capacity, such as time worked. Several studies have reported that firefighters experience heart rates close to maximum value [18] and a rapid onset of blood lactate accumulation [19]. The cardiorespiratory strain at such intensities creates a significant ACRE risk factor for those firefighters with low levels of fitness [12].

The efficacy of using sFSAs to classify ACRE risk has not been previously reported. Further insight is warranted to gain a better understanding of the efficacy of sFSAs in determining both physical readiness and the risk of suffering an ACRE. Therefore, the purpose of this retrospective analysis was to determine if there was a relationship between sFSA performance and ACRE risk in firefighters. A secondary purpose was to determine the effect that estimated VO_2_max has on sFSA performance. First, it was hypothesized that there would be a significant trend between performance classification (fast, moderate, and slow) and ACREs. Second, it was further hypothesized that estimated VO_2_max would significantly differ between sFSA performance groups. Finally, it was hypothesized that the estimated VO_2_max would be significantly correlated to sFSA performance.

## 2. Materials and Methods

The study was a retrospective design using archived data. The data were acquired from the participating fire department. The participating fire department was a medium-sized metropolitan fire department in the United States. The fire department operates 30 fire stations and responds to nearly 412,000 permanent residents. For inclusion in the analyses, a case (i.e., firefighter) had to meet the following inclusion criteria: (1) had to have completed the fire department’s physical ability test in 2015 or 2016 and (2) not be a duplicate case in the 2016 data set (i.e., the same firefighter). Cases were excluded if they were not males due to the limited number of females (n = 16) contained in the data set acquired from the participating fire department. The final data used in the analyses consisted of male (N = 524) firefighters. All firefighters were 18+ years of age.

The data were obtained from three sources: annual fitness assessments, annual physical ability tests, and department injury surveillance reports. Information collected from the fitness assessments included anthropometric measures (e.g., height, weight, and body fat percentage), estimated VO_2_max, muscular strength, muscular endurance, and flexibility. The physical ability test required each firefighter to complete five timed sFSAs: hose drag, search maze, rescue, forcible entry, and ladder raise and extension. Each test was separated by a 2 min rest period. Each test was individually timed in seconds. The times of the individual tests were summed to create a composite time in minutes. The sFSA performance variable was categorized into 3 levels of performance, using the 25th and 50th percentiles as a cutoff between fast (≤1.52), moderate (1.53–1.70), and slow (1.71+) performance levels [6,20]. Poplin et al. [6] and Lee et al. [20] used a similar approach to categorize performance data. Firefighters were allocated to a performance level based on their individual composite sFSA performance time. Estimated VO_2_max was the only metric from the annual fitness assessment used in the analyses. The procedures for conducting the estimated VO_2_max and the five sFSAs are described below.

### 2.1. Forestry Step Test

The forestry step test was used to measure aerobic capacity. The participant was required to step up and down on a 40-centimeter (cm) step/bleacher for five minutes at a rate of 22.5 steps·min^−1^ regulated by a metronome (90 beats per minute). The heart rate was measured for 15 seconds (s) during the time frame of 30 s post-exercise. Heart rate was measured with a standard fingertip pulse oximeter. This heart rate value was then compared to age-adjusted norms to determine a predicted VO_2_max value for each firefighter [21].

### 2.2. Simulated Fire Suppression Activities

#### 2.2.1. Hose Drag

The individual was required to pull a hose through a U-shaped course with several turns for a total of 30.48 meters (m). There was a ceiling on the U-shaped course to prevent the individual from standing upright. The event ended when the individual crossed over the end of the course with the nozzle end of the hose line. A stopwatch was used to record the length of time to complete the tasks.

#### 2.2.2. Search Maze

The individual was required to crawl through a dark wooden tunnel with obstructions and turns for approximately 24.08 m. The tunnel was 1.22 m high and 1.22 m wide. At one location in the tunnel, there was an obstacle on the floor from the ceiling. In addition, at two locations, the tunnel was reduced from 1.22 m to 0.91 m in width. The event ended when the individual emerged from the opposite end of the tunnel. A stopwatch was used to record the length of time to complete the tasks.

#### 2.2.3. Rescue

The individual was required to drag an approximately 58.97-kilogram (kg) dummy along a zigzag course to a designated area at the end of the course. There was a low ceiling over the course to prevent the individual from standing upright. The individual was not allowed to go outside the designated course. A stopwatch was used to record the length of time to complete the tasks.

#### 2.2.4. Forcible Entry

The individual was required to strike a rubber pad mounted on a movable post. A 3.18 kg sledgehammer was used to move the post a set distance. The post and structure were weighted to simulate the force needed to exert on a door to gain entrance. The individual’s score was based on the time taken to move the post the required distance of 17.78 cm. A stopwatch was used to record the length of time to complete the tasks.

#### 2.2.5. Ladder Raise and Extension

The individual was required to move a regulation ladder from a rack, rotate with the ladder, and carry the ladder a specified distance before laying it on the ground. The individual then moved to a second piece of equipment that was designed to simulate a ladder raise. The ladder had to be raised and a pulley was lowered (approximately 20.41 kg) in a hand-over-hand motion. Finally, the individual had to pick up the ladder on the ground and return it to the rack from which it was taken. The event ended when the ladder was returned to the rack. A stopwatch was used to record the length of time to complete the tasks.

### 2.3. Injury Defined

All data were merged with injury surveillance and de-identified by the participating fire department. The data were merged utilizing unique identifiers (e.g., C1) which enabled a direct comparison of persons with and without casualty. The “injury” episode was defined as any ACRE suffered by a firefighter requiring him to report the injury through the Tulsa Fire Department’s Injury Hotline or immediately to the supervising officer on the fireground. The researchers gained approval from the university institutional review board to conduct the retrospective analysis on the de-identified data set.

### 2.4. Statistical Analysis

The Cochran–Armitage trend test was run to determine whether a linear trend exists between the sFSA performance level and the proportion of firefighters who suffered an ACRE. The dependent variable was ACREs (YES/NO) occurring within 180 days of the firefighter’s physical ability test date. The independent variable was the sFSA performance level (fast, moderate, or slow). The differences in estimated VO_2_max between performance groups were evaluated using a one-way ANOVA. Spearman’s rho was conducted to determine the relationship between the estimated VO_2_max and sFSA performance. The alpha level was set a priori at 0.05. Statistical analyses were conducted using SPSS Statistics 28 (IBM Inc., Armonk, NY, USA).

## 3. Results

The proportion of firefighters suffering ACREs within each sFSA performance group is described in Table 1. In the cohort, 23 (4.4%) of the 524 firefighters suffered an ACRE within 180 days of their physical ability test. The sFSA performance levels were ≤1.52 min (n = 187), 1.53–1.70 min (n = 155), and ≥1.71 min (n = 159), and the proportions of patients suffering an ACRE were 2.1%, 4.3%, and 7.0%, respectively (Table 1). The Cochran–Armitage trend test showed a statistically significant linear trend, *p* = 0.023, with higher sFSA performance times associated with a higher proportion of firefighters going on to suffer an ACRE within 180 days of performing the sFSA.

A one-way ANOVA was conducted to determine if estimated VO_2_max differed between performance groups, earlier identified as fast (n = 168), moderate (n = 153), and slow (n = 169) (Table 2). Sample size differed in the between-group comparison of estimated VO_2_max, as compared to the number in each group identified in Table 1, due to missing participant data. There was homogeneity in the variances, as assessed by Levene’s test of homogeneity of variances (*p* = 0.181). Estimated VO_2_max was statistically significantly different between different performance groups, with F(2, 489) = 1150.56 and *p* < 0.001. Bonferroni post hoc analysis revealed estimated VO_2_max significantly (*p* < 0.001) decreased from the fast (M = 47.70 and SD = 7.78) to the moderate (M = 44.42 and SD = 7.47) groups. Estimated VO_2_max was also significantly (*p* < 0.001) higher in the fast group compared to the slow group. There was not a significant (*p* = 0.70) difference in estimated VO_2_max between the moderate (M = 44.42 and SD = 7.47) and slow groups (M = 42.53 and SD = 7.09). Spearman’s rho revealed that there was a significant negative correlation between estimated VO_2_max and sFSA performance, with r_s_(488) = −0.272 and *p* < 0.001.

## 4. Discussion

The purpose of this retrospective analysis was to determine if there was a relationship between sFSA performance and ACRE risk with a secondary purpose of exploring the role that estimated VO_2_max has on sFSA performance. The main findings were that there was a significant association between sFSA performance and ACREs. Firefighters within the moderate and slow performance classifications had two and four times higher proportions of injuries, respectively, as compared to fast performers. Additionally, it was observed that firefighters in the fast sFSA performance classification not only had a lower proportion of ACREs, but also greater aerobic fitness as compared to firefighters in the moderate and slow groups.

We hypothesized that there would be a significant trend between performance classification (fast, moderate, and slow) and ACREs. This hypothesis was supported. There was a significant linear trend between performance categories and ACREs, with the proportion of injuries increasing with poorer performance. This was the first study to explore the relationship between sFSA performance and ACREs. Several earlier studies have explored the influence that generalized components of fitness (e.g., estimated VO_2_max and other American College of Sports Medicine components of fitness) have on musculoskeletal injury; however, the relationship between performance and ACREs has received little attention. As it relates to musculoskeletal injury, investigators [7] have observed that individuals with a lower comprehensive fitness status had an increased risk of injury as compared to the most fit individuals. According to prior research, the risk of injury, such as sprains and strains, was 2.90 (95% CI 1.48–5.66) times more likely for the least fit individuals [7]. The findings of the present study suggest that aerobic fitness also plays a role in the incidence of ACREs, with moderate and slow performance fire classification having two and four times higher proportions of injuries, respectively, as compared to fast performers.

Although the number of musculoskeletal injuries is staggering in the firefighter population and more common than ACREs, the occurrence of ACREs during or after FSA presents a life-threatening emergency, with cardiac death being the stated cause of death in at least 17 of 96 firefighter fatalities in 2022. As reported in a systematic review [22] between the years 1990 and 2014, the percentage of fatalities attributed to sudden cardiac events consistently outnumbers the deaths due to burns or asphyxiation. Acute cardiac events are more likely to occur during or after FSAs as compared to other types of work-related duties, resulting in a 10-to-100 times greater risk of sudden cardiac events after FSAs versus station duties [13,22]. This is surprising given that firefighters spend only 1–5% of their duty time engaged in FSAs [13].

There are multiple interacting factors contributing to the heightened risk of ACREs during FSAs at the fireground, which include sympathetic nervous system activation, strenuous physical work, and exposure to intense thermal conditions and pollutants contained in fire smoke [22]. Firefighters who maintain adequate cardiorespiratory fitness and healthy body composition may be able to mitigate the effects of the first two factors (i.e., sympathetic nervous system activation and strenuous physical work) [12,18,22]. Relative VO_2_max is associated with systolic blood pressure, diastolic blood pressure, non-fasting blood glucose, and total cholesterol in firefighters [23,24]. Additionally, poor cardiovascular health is associated with relative cardiorespiratory fitness [23,24], which is associated with firefighter body composition. In a systematic review and meta-analysis [24], it was reported that obese firefighters had reduced cardiorespiratory fitness, and many did not meet the 42 mL/kg/min recommendation set forth by the National Fire Protection Association for active duty [11].

We hypothesized that estimated VO_2_max would significantly differ between sFSA performance groups. We further hypothesized that estimated VO_2_max would be significantly correlated to sFSA performance. Both hypotheses were supported by our findings and are consistent with previously reported research [25,26,27,28,29]. Skinner et al. [28] observed that in a cohort of aviation rescue firefighters, performance time on a simulated aviation rescue emergency protocol was inversely correlated to VO_2_max. This was also something reported by Marcel-Millet et al. [29], who observed that aerobic capacity and maximal strength were the strongest predictors of sFSA performance. In fact, the higher performers of sFSAs within the current study, on average, had higher cardiovascular fitness (as determined by estimated VO_2_max). The fast sFSA performance group demonstrated significantly higher estimated VO_2_max values as compared to the moderate and slow groups. The higher estimated VO_2_max may partly explain the lower proportion of ACREs in the fast sFSA performance group. While not significantly higher, on average, the moderate group had higher estimated VO_2_max values and a lower proportion of ACREs as compared to the slow group, which further suggests the benefits of cardiorespiratory fitness. Overall, the average estimated VO_2_max of each group was above the 42 mL/kg/min recommendation proposed by the National Fire Protection Association for optimizing cardio-protection in firefighters [7]. However, the slow sFSA performance group only exceeded the value by 0.53 mL/kg/min.

### 4.1. Limitations

The following limitations are acknowledged. First, the study was retrospective using archived data; thus, the original intent of the data was not to explore these trends. Because this was a retrospective study of archived data, the authors of the present study were not able to control for extraneous variables in the study design, which may have influenced the outcomes of the study. These limitations are especially noted for the testing procedures outlined for the sFSAs; however, the description of each test was taken directly from the physical ability test manual provided by the participating fire department. If authors had questions about test administration, the authors of the present study consulted with the individuals at the participating department tasked with facilitating the test battery.

Second, the data only consisted of male firefighters. As a result, our data cannot be generalized to female firefighters. According to available data [30], in 2014, nationally, there were an estimated 82,550 female firefighters. Between the years 2010 and 2014, overexertion or strain was the number one cause of work-related injury in volunteer and career female firefighters, accounting for 30% and 22% of injuries, respectively [30]. Future research to include female firefighters is warranted.

### 4.2. Recommendations

The relationship between cardiovascular health and cardiovascular fitness has been previously established [13,14] and findings from this current study demonstrate a relationship between ACREs and cardiovascular fitness. While the relationship was not directly tested in this study, it could be assumed that increases in cardiovascular fitness could reduce the risk of ACREs in active firefighters, given that estimated VO_2_max values differed across sFSA performance groups. Implementing a training program with cardiovascular fitness as one of the key components could assist in reducing the risk of ACREs in firefighters.

Findings from this study could also be used as a first step towards developing an effective screening tool for ACREs. A risk assessment for ACREs could be established using the three sFSA performance levels (fast, moderate, and slow) used during this study. Those who perform on the low end could be at a greater risk for ACREs and should undergo detailed medical screening to determine risks for ACREs and possibly duty status. While this study established a relationship between the sFSA performance and ACREs, a cause-and-effect relationship has yet to be established. Future research should examine the cause-and-effect relationship between the two variables to better establish a link between the sFSA performance and ACREs. Coupled with the current findings, more in-depth statistical analyses through predictive modeling could allow for the development of a more sensitive screening tool for ACREs.

## 5. Conclusions

In career firefighters, poorer sFSA performance is associated with a higher proportion of ACREs. The estimated VO_2_max values across sFSA performance levels did significantly differ, with the faster performers having better cardiovascular fitness. The results suggest that increasing cardiovascular fitness would improve sFSA performance and thus perhaps reduce the risk of ACREs.

## Figures and Tables

**Table 1:** Contingency table (2 × 3).

	sFSA Performance Levels			
ACRE	Fast (≤1.52 min)	Mod (1.53–1.70 min)	Slow (1.71+ min)	Total
NO	187	155	159	501
	97.9%	95.7%	93.0%	95.6%
YES	4	7	12	23
	2.1%	4.3%	7.0%	4.4%

Note. ACREs, acute cardiac and respiratory events; sFSAs, simulated fire suppression activities.

**Table 2:** Descriptive analysis of demographic, anthropometric, and motor parameters of study subjects.

	sFSA Performance Levels								
	Fast (≤1.52 min)			Mod (1.53–1.70 min)			Slow (1.71+ min)		
	N	Mean	SD	N	Mean	SD	N	Mean	SD
Age (years) *ƚǂ	191	34.54	7.33	162	37.35	6.85	171	41.86	7.58
Height (cm)	191	179.78	6.47	162	179.23	6.34	169	179.71	8.28
Weight (kg) *ƚǂ	191	89.29	11.84	161	93.62	14.13	171	100.73	17.91
Body Fat (%) *ƚǂ	190	12.91	3.73	161	15.39	4.19	171	17.47	4.12
Est. VO_2_max (mL/kg/min) *ƚ	168	47.70	7.78	153	44.42	7.47	169	42.53	7.09
BP Est. 1-RM (kg) ƚ	164	113.48	25.28	148	109.86	29.29	163	104.40	25.10
Relative BP Strength (%) *ƚǂ	164	1.28	0.28	147	1.19	0.30	163	1.06	0.27
Sit & Reach (cm)	190	46.51	8.58	161	44.81	7.62	170	44.67	8.01

Note. sFSAs, simulated fire suppression activities; mod, moderate; min, minutes; body fat %, estimated using 7-site skinfold measurement method; Est., estimated; BP, bench press; relative BP strength (est. 1RM/body mass); * *p* < 0.05, fast vs. moderate; ƚ *p* < 0.05, fast vs. slow; ǂ *p* < 0.05, moderate vs. slow.

## Data Availability

The datasets presented in this article are not readily available because the data are part of an ongoing study. Request to access the datasets should be directed to the corresponding author.
